# Tuberculosis treatment and Smoking, Armenia, 2014–2016

**DOI:** 10.1016/j.jctube.2017.04.001

**Published:** 2017-04-13

**Authors:** Dikran Raffi Balian, Karapet Davtyan, Andre Balian, Anna Grigoryan, Armen Hayrapetyan, Hayk Davtyan

**Affiliations:** a Tuberculosis Research and Prevention Center Non-Governmental Organization, 33/38 Charents Str., Nor Hachn, 2412, Armenia; b National Tuberculosis Control Center of Ministry of Health of Armenia, 10 Arzni Highway, Abovyan, 2201, Armenia

**Keywords:** Tuberculosis, Smoking, Treatment outcome, Treatment success

## Abstract

**Introduction:**

Tuberculosis and tobacco prove to be increasingly apparent world problems. Armenia is a developing country which is facing issues related to the high rates of tobacco consumption. Moreover, it is among the list of high multi-drug resistant (MDR) Tuberculosis TB burden countries. Treatment success rate in Armenia for sputum smear-positive cases never reached World Health Organization's (WHO) target of 85% in last 15 years. Data from different studies completed across the world suggests that there is an association between smoking and negative treatment outcomes.

**Methods:**

This retrospective study was designed to investigate aforementioned associations between TB treatment outcomes and smoking status of TB patients. Data for the study were derived from the national data available in the electronic database of the Armenian National TB Center.

**Results:**

Based on inclusion and exclusion criteria 992 TB patients registered in 2014 were enrolled in this study. All of them are were TB patients in which 387 were smokers and 605 were non-smokers. Notably, adjusted analysis showed that individuals who smoked during TB treatment had 1.61 higher odds of having unsuccessful TB treatment outcome. Additionally, consistent with the literature, statistically significant association was identified between TB treatment outcome and other well factors such as sputum smear status (OR = 2.24, *p* < 0.01), HIV status (OR, = 1.87, *p* < 0.01) of patients, etc.

**Conclusions:**

The smoking, HIV positive status, positive sputum smear microscopy test were identified as an important factors associated with the unsuccessful TB treatment outcome in Armenia. It highlights the necessity of having specific restrictions and campaign programs to reduce smoking rates among TB patients in order to improve current TB treatment and care services throughout Armenia.

## Introduction

Tuberculosis (TB) is proven to be a major public health problem in the world and particularly in Armenia. In general, among 100,000 individuals, 133 people are estimated to be contracted TB according to World Health Organization's (WHO) in 2014 [1]. With TB ranking alongside HIV as one of the leading cause of death in 2014 (1.5 million deaths), TB still proves to be an important subject to be studied [1]. Having negative TB treatment outcomes is considered to be one of the major causes of Drug Resistant TB which requires prolonged treatment with second line TB drugs and has lesser chances to be successfully treated [2]. Due to the high prevalence of drug-resistant(DR) forms of TB Armenia was included in the list of 27 countries with high multi-drug resistant (MDR) Tuberculosis TB burden [1]. In Armenia treatment success rate of Sputum Smear-Positive pulmonary TB is remaining to be below the WHO's target of 85% since 2001 [R]. In fact, Armenia's TB treatment success rate was 79.2% for TB cases registered in 2014. In Armenia, TB case detection rate is estimated to be 98% [R]. High case detection rate is essential for prevention as according to WHO if the disease goes untreated, each person with active TB infects roughly 10–15 other individuals every year [1].

On the other hand, Armenia is plagued by tobacco use. As per 2014 Health System Performance Assessment (HSPA) data, 58.1% of males and 3.5% of females are using tobacco products in Armenia, however, in the adult population, only 18.2% of adults do not smoke and are not exposed to secondhand smoke in the workplace. 43.9% of women are receiving second hand smoke in their homes from another family member [3]. In 2010, 96% of male smokers reported having smoked 10 or more cigarettes in the last 24 h [4].

Tsai et al. found that TB tended to be associated with cigarette smoking and other unhealthy lifestyle choices before TB diagnosis [5]. Jeyashree et al. explained that active smoking increases the risk of TB infection to 2–2.5 times and is significantly associated with recurrent TB and TB mortality [6]. Additionally, Ariyothai and colleagues studied cigarette smoking and its relation to pulmonary tuberculosis and found that the age of smoking initiation had a direct impact on TB. Smokers who started smoking at age 15–20 years old had a higher risk of Pulmonary TB compared to non-smokers. Even individuals who received second hand smoking from a family member were put at high risk for Pulmonary TB compared to individuals who are not exposed to secondhand smoking [7]. All these authors conducted their research in similar socio-economic areas that mirror Armenia's. Results in Armenia would be expected to yield similar results to these authors. Ferrara et al. found that smoking represents an important risk factor for TB, showing a trend between all aforementioned research about smoking and TB [8]. Leung et al. show that smoking adversely affects baseline disease severity, bacteriological response, treatment outcome and relapse in tuberculosis, further data shows that smoking cessation will likely reduce relapses and secondary transmissions [9]. As per treatment outcomes, data from Khan et al. show that in a study in Pakistan featuring 472 TB patients from whom 68 were smokers. 54 of the smoker patients had unsuccessful treatment results; the data analysis outcomes showed that there was statistically significant association between smoking and TB treatment outcomes [10].

Knowledge of smoking status associated with TB treatment outcomes might help efforts to improve the performance and quality of the TB services, but this has never been assessed in Armenia. We are interested in this topic to determine whether or not smoking has an effect on the outcomes of TB treatments.

## Methods

### Design

This was retrospective cohort study, using data from TB patients that began treatment in 2014.

### Setting

Armenia has 12 regions with rural and urban areas, including the capital region Yerevan. The National TB Program (NTP) provides TB care free of charge across Armenia. The DOTS (directly observed treatment, short-course) strategy was introduced in 2002 and has been used since. The introduction of TB outpatient clinics began in 2006; their locations being decided upon population covered and geographic criteria [11]. TB management follows WHO guidelines in Armenia with standardized treatment outcomes and include cured, treatment completed, died, lost to follow-up, failure and not evaluated [11].

### Inclusion criteria

All new presumed drug-susceptible TB patients, registered in 2014 within NTP in Armenia. Regular specifies that they have no form of drug-resistant tuberculosis.

### Exclusion criteria

TB patients with missing information on smoking status, re-treatment TB cases and DR TB patients were excluded from the study.

### Data collection, sources, and statistical analysis

The study related data was extracted from the national TB electronic database. All analysis had been performed using STATA 10.1 statistical software. Chi square test were used to test for differences between groups for all categorical variables and Student's *t*-test was used to test differences between groups for continues variables. The normality of the distributions was tested using Kolmogorov-Smirnov test. Multiple Logistic Regression analysis were used for adjusted analyses. Statistical significance was set at *p* = 0.05 (95% confidence interval) for all analyses.

### Ethics

The study protocol was approved and Ethics approval was obtained from the National TB Control Center of Ministry of Health of Armenia.

## Results

### Descriptive analyses

In Armenia 1022 new TB cases were registered in 2014. For 992 (97.1%) cases, data on smoking status was available and those 992 cases were selected as a study population. The mean age of the study population was 42.0 year. The study population consisted of 742 (74.8%) males and 250 (24.2%) females. Of the study population, 224 (22.6%) individuals tested positive for Sputum Smear microscopy test, while 768 (77.4%) tested negative. Mentioned 768 patients were diagnosed either by culture test or X-ray examination. The type of TB according to the anatomical site of disease among the study population consisted of 751 (75.7%) cases of Pulmonary TB, and 241 (24.3%) cases of Extra-Pulmonary TB. 70 (8.4%) subjects of the population were HIV+, however the HIV status of 159 (16.0%) people out of all study population were missing in the data. The treatment outcomes of the study population resulted in 99 (10.0%) cured, 637 (64.2%) treatment completed (736 (74.2%) success), 48 (4.8%) died, 12 (1.2%) treatment failed, 99 (10.0%) lost to follow-up, and 41 (4.1%) were not evaluated. Of the patients evaluated, 400 (40.3%) patients were smokers before TB treatment, and of that 400, 13 (3.3%) patients only quit during the TB treatment. 592 (59.7%) Patients were non-smokers prior to TB treatment, and no patients picked up smoking during treatment. 142 (14.1%) patients were using alcohol prior to treatment, and 135(13.6%) individuals remained alcohol users during treatment (Table 1).

**Table 1:** Demographic and clinical characteristics of patients with TB, Armenia, 2014–2016.

Variables:	Total n = 992 (%, mean ±SD)	Non-smokers n = 605	Smokers n = 387
**Demographics**			
**Age**	42.0 (±17.5)	39.1(±19.0)	46.6 (±13.7)
**Gender:**			
**Male**	742 (74.8)	361(59.7)	381(98.4)
**Female**	250 (25.2)	244(40.3)	6 (1.6)
**Clinical characteristics**			
**Sputum smear at diagnosis:**			
**+**	224 (22.6)	124 (20.5)	100 (25.8)
**-**	768 (77.4)	481 (79.5)	287 (74.2)
**Anatomical site:**			
**Pulmonary**	751 (75.7)	417 (68.9)	334 (86.3)
**EP**	241 (24.3)	188 (31.1)	53 (13.7)
**HIV status:**			
**+**	70 (8.4)	32 (6.3)	38 (11.8)
**-**	763 (91.6)	478 (93.7)	285 (88.2)
**Treatment outcome:**			
**Successful outcome**	736 (74.2)	482 (79.7)	254 (65.6)
***Cured***	*99 (10.0)*	*54 (8.9)*	*45 (11.6)*
***Treatmentcompleted***	*637 (64.2)*	*428 (70.7)*	*209 (54.0)*
**Unsuccessful outcome**	256 (25.8)	123 (20.83)	133 (34.4)
***Died***	*48 (4.8)*	*18 (3.0)*	*30 (7.8)*
***Treatmentfailed***	*12 (1.2)*	*8 (1.3)*	*4 (1.0)*
***Lost tofollowup***	*99 (10.0)*	*47 (7.8)*	*52 (13.4)*
***Notevaluated***	*97 (9.8)*	*50 (8.3)*	*47 (12.1)*
**Smoking status prior to TB treatment:**			
**Yes**	400 (40.3)	13 (2.1)	387 (100.0)
**No**	592 (59.7)	592 (97.9)	0 (0.0)
**Smoking status during TB treatment**			
**Yes**	387 (39.0)	0 (0.0)	387 (100.0)
**No**	605 (61.0)	605 (100.0)	0 (0.0)
**Alcohol consumption prior to TB treatment:**			
**Yes**	142 (14.3)	16 (2.6)	126 (32.7)
**No**	848 (85.7)	589 (97.4)	259 (67.3)
**Alcohol consumption during TB treatment:**			
**Yes**	135 (13.7)	13 (2.2)	122 (31.7)
**No**	854 (86.3)	591 (97.8)	263 (68.3)

### Unadjusted analysis

The normality of the distributions was confirmed with the Kolmogorov–Smirnov test. Afterwards either Pearson's chi^2^ test or Student's *t*-test were used to check the significance level of the association between dependent (treatment outcome) and independent variables. All of our independent variables impacted the treatment outcome in a statistically significant way except for age. However, after adjusting, some factors have been found to not be significantly associated with the treatment outcome (Table 2).

**Table 2:** Unadjusted analysis of the demographic and clinical characteristics and TB Treatment Outcomes, Armenia, 2014–2016.

Variables:	Successful treatment outcome n = 739 (72.3%)	Unsuccessful treatment outcome n = 283 (27.7)	Odds ratio or mean difference	*p* value
**Demographics**				
**Age**	41.4 (±17.5)	43.4 (±17.1)	2.0 (±1.2)	0.10
**Gender:**				
Female*	204 (27.6)	47 (16.6)	1.0	
**Male**	535 (72.4)	236 (83.4)	1.9	<0.01
**Clinical characteristics**				
**Sputum smear:**				
**SS +**	133 (18.0)	102 (36.0)	2.6	
SS -*	606 (82.0)	181 (64.0)	1.0	<0.01
**Anatomical site:**				
**Pulmonary**	541 (73.2)	237 (83.7)	1.9	
**EP**	198 (26.8)	46 (16.3)	1.0	<0.01
**HIV status:**				
**+**	45 (7.0)	29 (14.0)	2.2	
-*	595 (93.0)	178 (86.0)	1.0	<0.01
**Smoking status prior to TB treatment:**				
**Yes**	263 (35.7)	137 (53.5)	2.1	<0.01
No*	473 (64.3)	119 (46.5)	1.0	
**Smoking status during TB treatment:**				
**Yes**	254 (34.5)	133 (52.0)	2.1	<0.01
No*	482 (65.5)	123 (48.0)	1.00	
**Alcohol consumption prior to TB treatment:**				
**Yes**	84 (11.4)	58 (22.7)	2.3	<0.01
No*	652 (88.6)	197 (77.3)	1.00	
**Alcohol consumption during TB treatment:**				
**Yes**	80 (10.9)	55 (21.6)	2.3	<0.01
No*	655 (89.1)	200 (78.4)	1.0	

* Reference group

### Adjusted analysis

For adjusted analyses, potential confounders available in the data (age, gender, anatomical site and alcohol consumption), along with statistically significant variables were included into the multiple logistic regression model (Table 3). In the adjusted analysis compared to crude analysis odds ratio for Sputum Smear Microscopy result has changed from 2.6(*p* < 0.01) to 2.2 (*p* < 0.01), meaning that TB patients with positive result have 2.2 times higher odds of having unsuccessful treatment outcome. Additionally, odds ratio for HIV status has changed from 2.2 (*p* < 0.01) to 1.9 (*p* = 0.02), meaning that TB patients living with HIV have 1.9 times higher odds of having unsuccessful treatment outcome. Similarly, odds ratio for smoking status during the treatment changed from 2.1(*p* < 0.01) to 1.6(*p* = 0.02), indicating that those patients who smoked during treatment had 1.61 times higher odds for not succeeding the treatment. No other factors were significantly associated with treatment outcome in adjusted analyses.

**Table 3:** Adjusted logistic analysis of demographic and clinical characteristics and unsuccessful TB treatment outcomes, Armenia, 2014–2016.

	Odds ratio	CI	*p* value
**Age**	1.0	0.99–1.02	0.27
**Male gender**	1.4	0.86–2.19	0.19
**(+) Sputum smear**	2.2	1.51–3.31	0.00
**Anatomical site (pulmonary)**	0.8	0.50–1.30	0.38
**Positive HIV status**	1.9	1.09–3.22	0.02
**Smoking status during TB treatment**	1.6	1.07–2.42	0.02
**Alcohol consumption during TB treatment**	1.4	0.89–2.34	0.14
**Constant**	0.3	0.09–0.73	0.01

## Discussion

This first study aiming to evaluate the association between smoking and TB treatment outcomes has shown that there is statistically significant association and TB smoking problem among TB patients might need special attention.

Study identified sputum smear microscopy result, HIV status, and smoking during TB treatment being associated with the treatment outcome. These results are consistent with other studies published in peer reviewed journals.

The study displays strong correlation between smoking and TB treatment outcome. The study tested 996 individuals, with a treatment failure rate of 25.8%, with 51.9% of those failures being smokers. Smoking during TB treatment had much higher odds ratio of having unsuccessful treatment outcome, being 2.1. This data shows the necessity of implementing a “no-smoking” policy amongst TB patients.

Chuang et al. in a case-control study conducted in Taiwan comprising 359 subjects in total found that among culture positive cases currently smoking subjects had the highest TB treatment failure rate of 33% compared to former smokers and non-smokers. Additionally, study showed that current smoking was associated with longer culture conversion duration and so with longer treatment duration [12].

In another study conducted by Mahishale et al. data of 2350 TB patients from India were analyzed. Study showed that smoking was significantly associated with the treatment success rates as well as relapse rates, which was highest (12.9%) for smokers, compared to the ex-smoking population (10.4%), and the non-smoker populations (4.3%). Patients who were smokers had 72.5% treatment success rate, compared to the 77.7% for patients who were ex-smokers and 92.4% for patients who never smoked. The adjusted odds ratio for having successful treatment outcome was 0.69 for current smokers and 0.71 for ex-smokers compared to never smoker group. The adjusted hazard ratio for having a relapse was 1.68 for current smokers and 1.43 for ex-smokers compared to never smokers. Similar to current study's findings, these results show that quitting or not smoking at all increases the chances for having successful treatment outcome and decreases relapse rates [13].

In another study conducted in Taiwan compiling data of 5567 subjects in total, Yen et al. found that the recurrence of the TB was significantly associated with current smoking >10 cigarettes a day (adjusted hazard ratio 2.04), homelessness (adjusted hazard ratio 3.75), current comorbidities (adjusted hazard ratio 2.66) and positivity of the sputum smear microscopy test (adjusted hazard ratio 2.27) [14].

Tachfouti et al. found that the rate of treatment failure was significantly associated with smoking (adjusted odds ratio 2.25) and monthly income (adjusted or 3.23). Sample size of the study was 1039 and subjects were selected from 15 different tuberculosis control units covering more than 60% of the Moroccan population [15].

In a research conducted in Brazil which studied 360 patients, Maciel et al. found that those who smoked at the time of diagnosis had 3.04 times higher odds of remaining culture positive after 2 months of treatment compared to non-smokers [16].

### Study strength

The strengths of the study are that data from all TB facilities in the country were included. This means that the study findings are coming from routine setting reflecting the real situation on the field. The missing data is about 3% showing generalizability of data. Also this study considered operational research priorities expressed in the NTP review report of Armenia [R].

### Study limitations

The main potential limitation of this study could be the bias existing in the obtained data, as there were no opportunities to verify it with the source documentation. There is a possibility of human error during data entry into the TB Database as well as there is a possibility of misclassification during initial recording into paper forms by doctors/nurses.

## Conclusions and recommendations

The smoking, HIV positive status and positive sputum smear microscopy test result identified as an important factors associated with the unsuccessful TB treatment outcomes in Armenia. It highlights the necessity of having specific restrictions and campaign programs to reduce smoking rates among TB patients in order to improve current TB treatment and care services throughout Armenia.

## Conflict of interest

No conflict declared.

## Funding

There was no funding available for this study. La Fondation Veuve Emile Metz-Tesch (Luxembourg) funded the cost of production of SORT IT online materials. Additional support was provided by WHO/TDR for covering the cost of publication in an open access journal. Funders had no role in study design, data collection and analysis, decision to publish, or preparation of the manuscript.
